# The world cancer patient population (WCPP): An updated standard for international comparisons of population-based survival

**DOI:** 10.1016/j.canep.2020.101802

**Published:** 2020-12

**Authors:** Adalberto Miranda-Filho, Freddie Bray, Hadrien Charvat, Swaminathan Rajaraman, Isabelle Soerjomataram

**Affiliations:** a Section of Cancer Surveillance, International Agency for Research on Cancer, Lyon, France; b Department of Epidemiology, Biostatistics and Cancer Registry, Cancer Institute (WIA), Chennai, India

**Keywords:** Age-standardised survival, Cancer epidemiology, Net survival, Standards

## Abstract

**Purpose:**

This study addresses the need for a global cancer patient-based standard population that adjusts for the expected age structure of different cancers, thus aiding the comparison of survival estimates worldwide.

**Methods:**

Counts of age-specific incidence for 36 cancer sites in 185 countries for the year 2018 were extracted from IARC’s GLOBOCAN database of national estimates. We used a multinomial mixture regression to identify clusters of cancer sites with similar age-specific profiles.

**Results:**

An updated standard entitled the World Cancer Patient Population (WCPP) is presented, derived from the current estimated global numbers of cancer patients that comprises three sets of age-specific weights. Around two-thirds of cancer sites were described by a unique standard, representing the majority of epithelial cancers more often diagnosed at older age groups. The two other standards represent a number of non-epithelial cancer types, and cancers common at younger and older age groups, respectively.

**Conclusion:**

The WCPP proposed here provides a contemporary and global means to estimate age-standardised survival for international benchmarking purposes.

## Highlights

• We propose an update of a global standard for cancer survival comparisons entitled the World Cancer Patient Population (WCPP), constructed from the current global age distributions of cancer patients.
• From an analysis of the 36 major cancer types, three standards of age-specific weights are derived to enable age-adjusted comparisons of cancer-specific survival.
• Around two-thirds of cancer sites were described by one standard, representing the majority of epithelial cancers more often diagnosed at older age groups.
• The two other standards represent a number of non-epithelial cancers that are more common among younger and older age groups, respectively.

## 1 Introduction

Population-based cancer survival is a key measure of the effectiveness of national health services [1], yet international comparisons of all-ages cancer survival estimates may be invalidated by varying risks of dying at different ages. Given that cancer patients in different populations have distinct age structures with different risks of death [2], direct age standardisation is commonly used to compare survival estimates across countries, adjusted for the confounding effects of age [3]. The first effort to develop a standard to estimate age-standardised relative survival was undertaken as part of IARC’s *Cancer Survival in Developing Countries* (SURVCAN) project in 1998 [4]. At the time, the first World Cancer Patient Population (WCPP) was proposed based on the global estimates of cancer incidence in the 1980s. Nevertheless, the previous version of WCPP was limited in the number of age groups and cancer sites assessed based on the earlier version of the International Classification of Diseases (ICD-9). As part of the EUROCARE project, a standard covering four cancer groups compiled from cancer patient data in 30 countries within Europe has often been used as an international standard subsequently.

While age-standardised cancer survival estimates are necessarily synthetic, there is a need for summary measures to be based on contemporary age-group distributions of cancer patients using the most recent disease classification (e.g. ICD-10). With an international expansion in the capacity for cancer registries to construct robust cancer survival estimates for benchmarking purposes, there is a need to review the age structure of cancer patients in the comparative assessments of age-standardised survival. This particularly the case in low-and middle income countries (LMIC), where the underlying age distributions of patients can be markedly different from high income countries. We thus propose an updated WCPP that captures the present global distribution of cancer patients and generates age-adjusted cancer survival estimates suitable for international geographic and temporal comparisons.

## 2 Methods

### 2.1 Data source and population

We use national estimates of cancer incidence for 36 types worldwide retrieved from GLOBOCAN 2018 produced by the International Agency for Research on Cancer and presented in the Global Cancer Observatory (GCO, http://gco.iarc.fr) [5]. The methods to estimate the global cancer incidence utilises the most reliable cancer incidence and mortality data at the national or subnational level, as explained elsewhere [6]. We used available estimates for 185 countries and 18 age groups (ages 0‐4, 5‐9, …, 80‐84, and >85 years), combining both sexes. Cancer sites were classified according to the International Classification of Diseases 10th revision (ICD-10: C00‐C97) into 36 cancer types.

### 2.2 Statistical models

For each of the 36 cancer sites, the proportion of cases by 5-year age group was calculated. Multinomial mixture modelling was then used to identify clusters of cancer sites with similar age distributions. The model assumes that the observed age distributions arise from a mixture of a finite number (to be determined) of component multinomial distributions; the model parameters are obtained by the expectation-maximisation algorithm. We applied the multinomial mixture model allowing the number of classes (cancer types) to vary between 1 –36 classes. We used the Bayesian Information Criterion [7] to determine the best fitting model that, in the first instance, partitioned cancer sites into five classes. One of these five classes represented a relatively rare group of cancers (brain and CNS, leukaemia, non-Hodgkin lymphoma, and salivary glands) with distinct bimodal age patterns observed, with peaks in children and in adults, while the corresponding distributions for oesophagus and stomach cancer appeared to fit two different classes (details provided in supplementary material). Thus, practical considerations led us to consider only models with at most three mixture components, with selection of the optimal number of components based on the Bayesian Information Criterion [8]. Finally, each cancer site was assigned to the component corresponding to the highest posterior probability of membership.

All analyses were performed with R statistical software version 3.3.3, with the *mixtools* package [9] used for finite mixture modelling.

## 3 Results

**Fig. 1:**
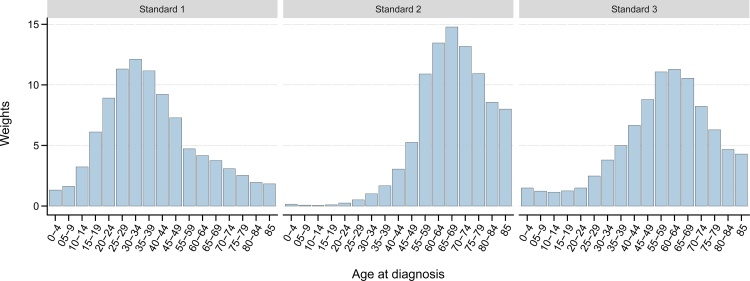
Weights of the three standards based on the age distribution of 36 cancer sites estimated in GLOBOCAN 2018.

Fig. 1 and Table 1 show the three standards of age-specific weights that comprise the World Standard Cancer Patient Population (WCPP), based on modelling of each of the 36 individual cancer types: standard 1 is representative of non-epithelial cancer sites frequently diagnosed at younger ages; standard 2 represents subsets of epithelial cancers occurring mainly in older adults, while standard 3 is in line with cancers frequently at older ages, but also at younger ages. Standard 1 gives greater weighting to patients aged from 25 to 45 years old (43.8 %), representing the age profiles of Hodgkin lymphoma, Kaposi sarcoma, and testicular cancer. Standard 2 represents two-thirds of cancers and comprises cancer types that are often diagnosed at older ages (55.4 % for those aged 65+), namely cancers of the anus, bladder, colon, corpus uteri, gallbladder, hypopharynx, kidney, larynx, liver, lung, oesophagus, oropharynx, pancreas, penis, prostate, rectum, stomach, vagina, and vulva, as well as melanoma of the skin, non-melanoma of skin, mesothelioma and multiple myeloma. Standard 3 represents cancers common in adults as well as children and/or young adult populations, including cancers of the brain and nervous system, breast, cervix uteri, lip, oral cavity, nasopharynx, ovary and salivary glands, as well as non-Hodgkin lymphoma and leukaemia.

**Table 1:** Estimated number of new cancer diagnoses and weights* by 5-year age group for the three standards of the world cancer patient population (WCPP). Source: GLOBOCAN 2018.

	Standard 1^a^		Standard 2^b^		Standard 3^c^	
Age	Number of patients	Weight	Number of patients	Weight	Number of patients	Weight
0−4	2654	1.32	12,648	0.15	62,882	1.10
5−9	3559	1.62	5434	0.07	50,019	0.97
10−14	6581	3.23	4169	0.05	43,054	0.96
15−19	12,129	6.11	8135	0.10	41,995	1.18
20−24	17,209	8.91	20,159	0.25	56,109	1.57
25−29	21,498	11.31	38,414	0.52	115,340	2.73
30−34	22,760	12.12	71,576	1.01	199,583	4.21
35−39	20,750	11.16	124,836	1.67	278,586	5.44
40−44	17,160	9.22	247,155	3.05	376,464	7.18
45−49	13,752	7.30	488,333	5.26	490,219	9.35
50−54	10,971	5.68	818,074	8.05	567,795	10.69
55−59	9306	4.71	1,189,485	10.91	595,446	11.21
60−64	8369	4.16	1,562,213	13.46	591,639	11.18
65−69	7553	3.75	1,831,412	14.78	545,112	10.23
70−74	6143	3.09	1,664,726	13.18	425,206	7.84
75−79	4919	2.53	1,380,864	10.93	320,646	5.91
80−84	3696	1.95	1,087,515	8.57	233,944	4.32
85+	3377	1.83	1,008,407	7.99	212,576	3.93
Total	192,386	100	11,563,555	100	5,206,615	100

* The numbers of patients reflect the number of cancer cases included in each standard. Weights represent the mixture of proportion of new cancer cases across age groups and cancer types.”.

a Standard 1: Hodgkin lymphoma (C81), Kaposi sarcoma (C46), and testis (C62).

b Standard 2: Anus (C21), bladder (C67), colon (C18), corpus uteri (C54), gallbladder (C23‐C24, incl. extrahepatic ducts), hypopharynx (C12‐C13), kidney (C64‐C65, incl. renal pelvis), larynx (C32), liver (C22, including intrahepatic bile ducts), lung (C33‐C34, incl. trachea and bronchus), melanoma of the skin (C43), non-melanoma skin cancer (C44), mesothelioma (C45), multiple myeloma (C88 and C90, incl. immunoproliferative diseases), oesophagus (C15), oropharynx (C09‐C10), pancreas (C25), penis (C60), prostate (C61), rectum (C19−20), stomach (C16), vagina (C52), and vulva (C51).

c Standard 3: Brain, central nervous system (C70‐C72), breast (C50), cervix uteri (C53), leukemia (C91‐C95), lip, oral cavity (C00‐C06), nasopharynx (C11), thyroid (C73), non‐Hodgkin lymphoma (C82‐C86 and C96), ovary (C56), and salivary glands (C07‐C08).

Supplementary Fig. 1 provides an empirical example based on observed survival data from 67 populations mostly in LMIC settings. Plotted as a boxplot, the ratios of the age-standardised 3-year net survival (age-adjusted using the WCPP) and the unadjusted 3-year net survival showed small variability for the vast majority of registries. This was seen for cancer sites age-standardised using standard 1 (testicular cancer), standard 2 (colorectal and prostate cancers) and standard 3 (breast cancer and leukaemia).

## 4 Discussion

Cancer survival is a key indicator of progress in cancer control at the population level, reflecting a complex set of system and host determinants including the effectiveness of cancer services, socioeconomic factors, and health-seeking behaviour [10]. In this study, we present an updated standard entitled the World Cancer Patient Population (WCPP), constructed from the most recent estimates of the global number of cancer patients, and comprising three standards of age-specific weights. The updated WCPP can be reasonably mapped to the age structure of most cancer forms, and thus serve the purpose of age-adjustment of cancer survival estimates that enables international benchmarking.

It should be noted that while the standard populations derived here produce synthetic age-adjusted estimates of survival, it is an essential adjustment for comparisons between regions with differing demographic and development characteristics. In the EUROCARE study [11], the age-structure of cancer patients from the European registries included was used, reflecting an age distribution of patients diagnosed in very high income countries that were commonly older than those in LMIC. It is evident that for survival benchmarking purposes in LMIC, the age structure requires a case distribution that better reflects the underlying age profiles observed in the concerned communities. The proposed WCPP gives greater weight to younger patients, but ultimately it enables age-standardised survival estimates to be compared across population worldwide, irrespective of their age structures.

There are some communalities with the world standard population proposed by Segi in 1960 [12], later modified by Doll [12] which gained universal acceptance as a means to compare age-adjusted rates of incidence in global comparative studies. Both age-adjusted incidence and relative survival are synthetic indicators, yet the latter is commonly taken at face value by clinicians, patients and policy makers as an indicator of cancer survival in individual cancer patients. In addition, the use of external standard cancer populations may lead to misinterpretation of net survival; changes in the patient age structure over time might lead to misleading conclusions when countries are compared according to rank or progress of cancer care over time [13]. An alternative approach to age standardisation has been proposed by Brenner and colleagues [13] that resolves these issues and was used in SurvCan-2 [9]. The method however requires individual-level weighting hindering external comparisons with published survival statistics. The main characteristic of direct age-standardisation is related to the gap between observed and age-adjusted values that may create inconsistencies that artificially change the rank of the survival estimates on comparing regions [3]. Nevertheless, bearing in mind its limitation, direct standardisation using external population continues to be an effective tool to present international comparisons between countries [5], although there remains a need for education as to the interpretation of such summary measures.

A final remark on the limitation of this study pertains to the source of data used to develop the WCPP, namely the incidence counts obtained at the national level from IARC’s GLOBOCAN database. These estimates are derived from the best available cancer data within a given country; population-based cancer registries where available, that either cover national populations, or, as is more common in many LMIC, cover one or more smaller, subnational catchment populations. As such the validity of the GLOBOCAN estimates depends largely upon the representativeness and quality of the source information. A full description of the methods applied in GLOBOCAN has been described elsewhere [14].

We reiterate that age-adjusted survival is useful for comparisons between populations and in a single population over time, as long as a common set of standard weights are applied. The use of age-adjusted survival using the WCPP produces estimates that are in line with the present global age distribution of cancer patients. Nonetheless, if the requirement is to present survival estimates that best reflect the clinical reality of a specific group of cancer patients, then non age-adjusted survival should be reported.

To conclude, we provide here a global standard for generating age-standardised survival estimates. The modelling process identified three standards covering a wide range of age profiles linked to the epidemiology, biology and natural history of the specific cancer types. This updated WCPP has been developed to enable international comparisons of survival, and is particularly relevant when LMIC are included in such benchmarking exercises.

## Disclaimer

Where authors are identified as personnel of the International Agency for Research on Cancer/World Health Organization, the authors alone are responsible for the views expressed in this article and they do not necessarily represent the decisions, policy or views of the International Agency for Research on Cancer/World Health Organization.

## CRediT authorship contribution statement

**Adalberto Miranda-Filho:** Conceptualization, Methodology, Software, Investigation, Writing - review & editing. **Freddie Bray:** Conceptualization, Supervision. **Hadrien Charvat:** Methodology, Visualization, Software, Investigation. **Swaminathan Rajaraman:** Conceptualization. **Isabelle Soerjomataram:** Conceptualization, Supervision, Writing - review & editing.

## Declaration of Competing Interest

The authors report no declarations of interest.
