# Supplementary material for: The world cancer patient population (WCPP): An updated standard for international comparisons of population-based survival
Source: Cancer Epidemiol. 2020 Dec;69:101802. doi: 10.1016/j.canep.2020.101802 (PMC7768180; doi:10.1016/j.canep.2020.101802)
Supplement: Supplementary file 2 [file mmc2.docx]

**Supplementary material**

**Title**: The world cancer patient population (WCPP): an updated standard for international comparisons of population-based survival

*Supplementary methods*

We applied the multinomial mixture model allowing the number of classes to vary between 1 to 36 classes. As the EM algorithm highly depends on the initial values (randomly chosen by the *multmixmodel* function), the solution was obtained using a fit criterion, namely the Bayesian Information Criterion (BIC). Other than for oesophageal and stomach cancer, each class contained those cancer sites with probabilities higher than 0.99. The supplementary Table 1 shows the best fit model constructed with five classes and the probability that each cancer type belongs to one of the five classes.

Class 1 includes a range of tumours with bimodal age patterns (brain and CNS, leukaemia, non-Hodgkin lymphoma, and salivary glands), while two rather common cancer types (stomach and oesophagus) presented probabilities that indicated membership of classes 3 and 4. We thus decided to reduce the number of classes to three, with brain and CNS, leukaemia, non-Hodgkin lymphoma, and salivary glands relocated to the new class 1 and stomach and oesophagus reallocated to group 2, with a probability higher than 0.99.

*Supplementary results*

Estimates of net survival were obtained for 67 population-based cancer registries, mostly in low- and middle-income settings, from the SURVCAN-3 project ([www.survival.fr/survcan](http://www.survival.fr/survcan)). Net-survival estimates using Pohar Perme method were calculated for: (*a*) 3-year age-standardised net survival calculated using the world cancer patient population (WCPP); (*b*) 3-year age-standardised net survival calculated using the EUROCARE standard (ICSS), and (*c*) unadjusted 3-year net survival. Thereafter we estimated the survival ratio calculated by dividing (*a*) by (*c*) and (*b*) by (*c*). The ratio values were plotted as boxplots for the following cancer sites: female breast (C50), cervix uteri (C53), colorectal (C18-20), leukaemia (C91-95), lung (C33-34), non-Hodgkin lymphoma (C82-86, 92), Ovary (C56), prostate (C61) and testis (C62).

Supplementary figure 1 shows the 3-year net survival ratios by cancer site using the two different sets of standards. Ratios close to unity signify that the age-standardised and unadjusted net survival estimates are similar. Overall, a few registries presented variations in the ratio higher or lower than 20%, using either the WCPP or EUROCARE standards. The WCPP and EUROCARE standards use different age weights and more marked differences were seen for breast, leukaemia, non-Hodgkin lymphoma and ovarian cancer. Larger variations may be expected where differences exist between the age group structures in the observed registry data, for example related to differences in the age onset of specific cancer types.

Supplementary Figure 1. Ratios of age-standardised values and unadjusted net survival estimates in selected population-based cancer registries worldwide using two different standards.

Footnote

NHL: Non-Hodgkin lymphoma. Registries included by cancer site in each boxplot are in parentheses: breast (63), cervix uteri (55), colorectal (54), leukaemia (51), lung (50), non-Hodgkin lymphoma (53), ovary (51), prostate (53) and testis (26).
